# Supplementary figures and images for: Using Ethereum blockchain to store and query pharmacogenomics data via smart contracts
Source: BMC Med Genomics. 2020 Jun 1;13:74. doi: 10.1186/s12920-020-00732-x (PMC7268467; doi:10.1186/s12920-020-00732-x)

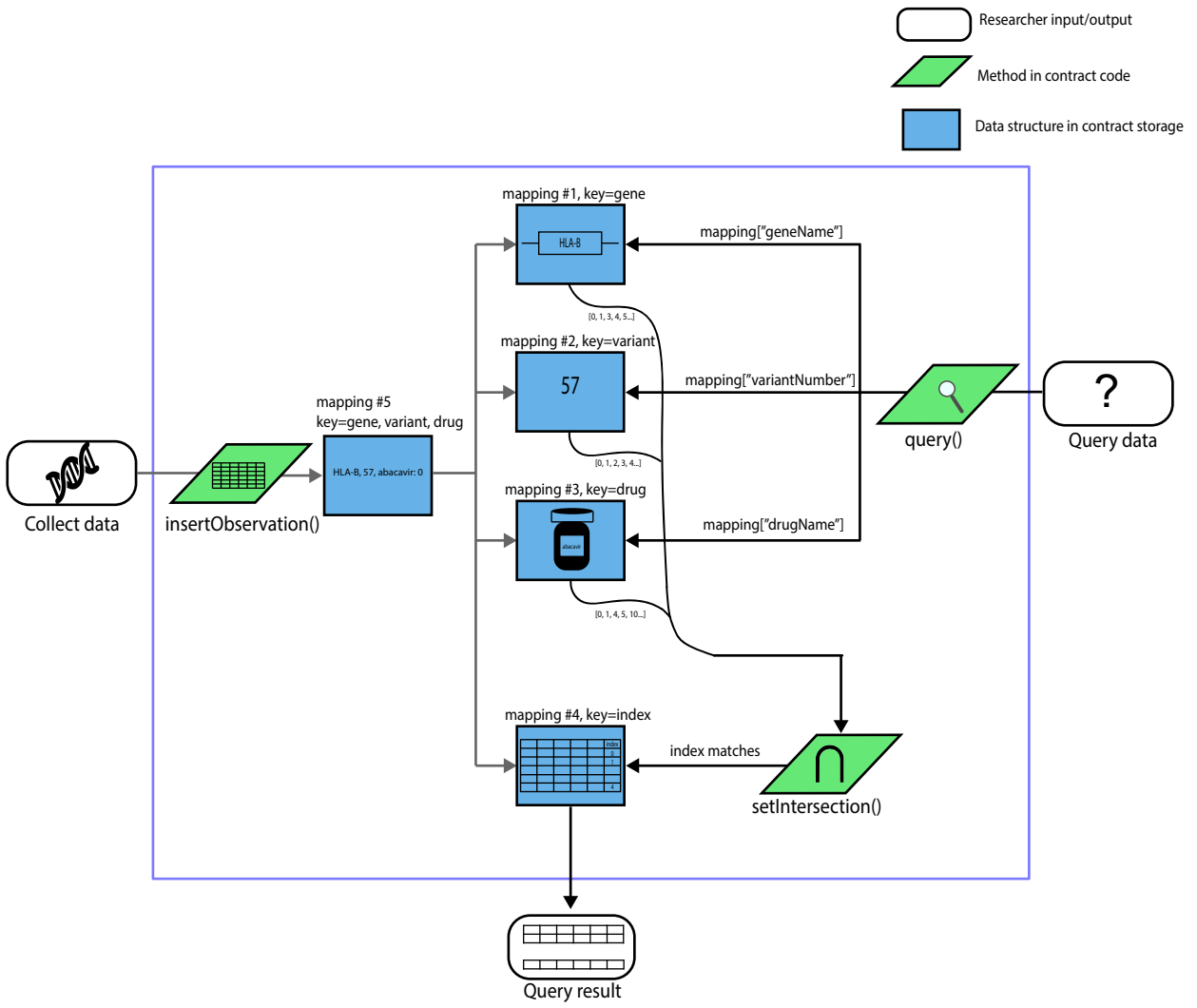

Supplement: Supplementary file 1 — Additional file 1 Supplementary Information. A pdf file including a flowchart showing the design of the fastQuery smart contract [file 12920_2020_732_MOESM1_ESM.pdf]
